# A Two-Layer Graphene Nonwoven Fabric for Effective Electromagnetic Interference Shielding

**DOI:** 10.3390/ma17153747

**Published:** 2024-07-29

**Authors:** Ying Wu, Haijun Tang, Liying Kang, Hongfu Li, Naisheng Jiang

**Affiliations:** School of Materials Science and Engineering, University of Science and Technology Beijing, Beijing 100083, China; b20200241@xs.ustb.edu.cn (H.T.); kangly@ustb.edu.cn (L.K.)

**Keywords:** two layer, graphene, nonwoven fabric, electromagnetic interference shielding

## Abstract

Rapid advancements and proliferation of electronic devices in the past decades have significantly intensified electromagnetic interference (EMI) issues, driving the demand for more effective shielding materials. Herein, we introduce a novel two-layer graphene nonwoven fabric (2-gNWF) that shows excellent EMI shielding properties. The 2-gNWF fabric comprises a porous fibrous upper layer and a dense conductive film-like lower layer, specifically designed to enhance EMI shielding through the combined mechanisms of reflection, multiple internal reflections, and absorption of electromagnetic waves. The 2-gNWF exhibits a remarkable EMI shielding effectiveness (SE) of 80 dB while maintaining an impressively low density of 0.039 g/cm^3^, surpassing the performance of many existing graphene-based materials. The excellent EMI shielding performance of 2-gNWF is attributed to the multiple interactions of incident electromagnetic waves with its highly conductive network and porous structure, leading to efficient energy dissipation. The combination of high EMI SE and low density makes 2-gNWF ideal for applications that require lightweight yet effective shielding properties, demonstrating the significant potential for advanced EMI shielding applications.

## 1. Introduction

With rapid technological advances, the proliferation of electronic devices has led to a significant increase in electromagnetic interference (EMI). This phenomenon involves the disturbance of electronic systems by external electromagnetic fields, which can critically impair the performance and reliability of electronic devices [1,2]. In addition, EMI radiations are detrimental to human health [3]. Effective EMI shielding is essential to protect sensitive equipment from such disturbances, ensuring their proper functionality and longevity. Traditional EMI shielding materials, such as metals, have been widely used due to their high electrical conductivity and effectiveness in blocking electromagnetic waves [4,5]; however, these materials are often associated with high density and limited flexibility, which restricts their application in modern electronics that demand lightweight, flexible, and compact designs [4,5]. Consequently, there has been a growing interest in developing new materials that can provide effective EMI shielding while overcoming the limitations of conventional metal-based shields.

To develop lightweight EMI shielding materials, researchers have incorporated various conductive fillers and networks—such as graphene, carbon nanotube, transition metal carbides and nitrides (MXene), metallic nanoparticles or nanowires, and liquid metal—into polymer matrices to obtain effective shielding composites [5,6,7,8,9]. Among these conductive materials, graphene, a two-dimensional carbon material, has emerged as a promising candidate for advanced EMI shielding applications [10]. Its exceptional electrical conductivity, mechanical strength, and low density make it an ideal material for creating lightweight and efficient shields [11,12]. Although polymers exhibit low densities, they are generally transparent to EMI radiations with extremely low EMI shielding effectiveness (SE) of smaller than 1 dB, making little contribution to the final EMI shielding properties of materials [13,14]. Though good EMI shielding properties have been achieved, the reduction of the densities of polymer-based composites is limited.

Alternatives, freestanding and porous conductive materials have been demonstrated to be particularly effective for lightweight EMI shielding [15,16]. For example, an enhanced EMI SE, or specifically absorption-induced SE (SE_A_), was obtained by foaming graphene paper despite the lower electrical conductivity, while the reflection-induced SE (SE_R_) showed little change [17]. Multiple reflections between conductive skeletons of the porous structure enhance interactions between the shielding materials and incident waves and therefore contribute to wave attenuation and absorption [18]. Taking advantage of graphene, numerous studies have explored various forms of porous graphene-based materials, such as reduced graphene oxide (rGO) films, pristine graphene films, graphene aerogels, and graphene foams, achieving good EMI shielding properties and low densities [19,20]. Despite these advancements, achieving high EMI shielding effectiveness while maintaining low density is still a pursuit of current studies.

In this study, we have developed a novel two-layer graphene nonwoven fabric (2-gNWF) that leveraged the excellent properties of graphene and a unique structural design to achieve enhanced EMI shielding performance. The 2-gNWF was fabricated by vacuum filtration of graphene oxide (GO) fiber fragments and reduction, resulting in a highly porous fibrous upper layer and a dense conductive film-like lower layer. This structure is designed to maximize the interactions with electromagnetic waves through reflection, absorption, and dissipation mechanisms. The 2-gNWF showed an absorption-dominated EMI SE of approximately 80 dB in the X-band frequency range while maintaining a remarkably low density of 0.039 g/cm^3^. We believe these findings will shed new light on the potential for widespread adoption of advanced graphene-based shielding materials across various high-tech industries, significantly contributing to the advancement of electronic technology and enhancing protection against electromagnetic interference.

## 2. Experimental Section

### 2.1. Fabrication of GO Fibers

GO fibers were fabricated via wet-spinning technology [21] by injecting GO dispersions in N, N′-dimethylformamide (DMF, Aladdin, Shanghai, China) solvents into an ethyl acetate (Aladdin) coagulating bath. The as-purchased GO aqueous dispersions (Gaoxi Tech., Hangzhou, China, http://www.gaoxitech.com) were conducted a solvent exchange process using DMF for three times, obtaining GO/DMF dispersions with a concentration of 6 mg/mL. This GO/DMF dispersion was degassed before adding in an injector, followed by spinning into the ethyl acetate coagulating bath via a capillary tube with a diameter of 0.18 mm. The as-spun GO fibers were dried before continuous collection using a cylindrical graphite rotator.

### 2.2. Preparation of 2-gNWFs

**Figure 1:**
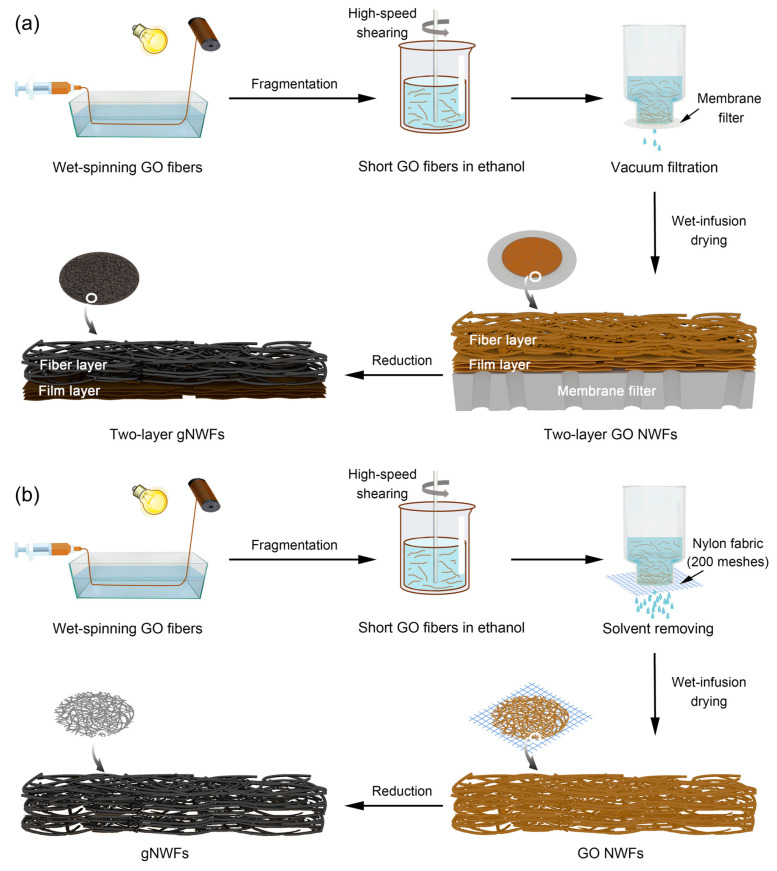
Schematic illustrations of the fabrication process of (**a**) two-layer gNWFs (2-gNWFs), and (**b**) gNWFs.

After annealing the as-spun GO fibers at 80 °C for 2 h in the atmosphere, 60, 80, 100, and 120 mg GO fibers were re-dispersed in ethanol and fragmented by a high-speed shear mixer with a speed of 10k rounds per minute for 1 min. The two-layer GO NWFs were prepared by filtrating fragmented GO fibers through the membrane filter (Anodisc, 47 mm in diameter, 0.2 μm in pore size, supplied by Whatman, Whatman Ltd., Maidstone, UK) under vacuum pressure. The two-layer GO NWFs were chemically reduced by hydroiodic acid (HI, Aladdin) solutions at 95 °C overnight, followed by rinsing with ethanol to remove HI acid residuals. The partially reduced two-layer NWFs were dried and further thermally reduced at 1600 °C for 1 h to obtain 2-gNWFs. The schematic process is shown in Figure 1a.

### 2.3. Preparation of gNWFs

The preparation process of gNWFs is similar to that of 2-gNWFs, except for the absence of vacuum filtration (Figure 1b). Specifically, 60, 80, 100, and 120 mg annealed GO fibers were re-dispersed in ethanol and fragmented by a high-speed shear mixer with a speed of 10k rounds per minute for 1 min. The GO fiber fragments were collected using a 200-mesh nylon fabric to remove ethanol. GO NWFs were obtained after infusion and drying, which were chemically reduced by HI acid solutions at 95 °C overnight and thermally reduced at 1600 °C for 1 h to obtain gNWFs.

### 2.4. Characterizations

The as-purchased GO dispersions were deposited on a silicon substrate with a 200 nm thick silicon dioxide layer. GO sheets were observed using a scanning electron microscope (SEM, Hitachi S4800, Hitachi Ltd., Chiyoda-ku, Japan), based on which the size distribution was analyzed with assistants of the software ImageJ V1.40. The length distribution of GO fiber fragments and the diameter distribution of fiber skeletons in 2-gNWFs and gNWFs were also calculated using ImageJ based on corresponding fabric photos. Morphologies of NWFs were observed by SEM. The electrical conductivity of both 2-gNWFs and gNWFs was measured using a two-point method, where a rectangular sample was applied. To ensure accurate measurements, the two ends of each sample were connected to copper wires using a silver paste, which served to minimize contact resistance and improve the reliability of the electrical measurements. The electrical conductivity (*σ*) was calculated based on the measured resistance (*R*):

(1) $$\sigma =\frac{L}{RS}$$

where *L* and *S* are the length and cross-sectional area of samples, respectively.

The scattering parameters (S-parameters), *S*_11_ and *S*_21_, were measured using a vector network analyzer (ZNB-40, Rohde & Schwarz, Munich, Germany) by a waveguide transmission line method in the X-band frequency range of 8.2–12.4 GHz. Samples with areas of 22.9 × 10.2 mm^2^ were placed between the waveguide tube and the adapter, before being fastened tightly to avoid microwave leakage. The total EMI SE is defined as the logarithm of the power ratio of the incident wave, *P*_I_, and the transmitted wave, *P*_T_ [13]:

(2) $${SE}_{T}(dB)=10\log\left(P_{I}/P_{T}\right)$$

where *P*_I_ and *P*_T_ can be determined by S-parameters, *S*_11_ and *S*_21_ [22]:

(3) $$\left|S_{11}\right|=\sqrt{P_{R}/P_{I}}$$

(4) $$\left|S_{21}\right|=\sqrt{P_{T}/P_{I}}$$

where *P*_R_ is the power of reflected waves. The normalized reflected power, *R*, the normalized transmitted power, *T*, and the normalized absorbed power, *A*, can be calculated by Equations (5)–(7) [23]:

(5) $$R=S_{11}^{2}$$

(6) $$T=S_{21}^{2}$$

(7) A=1−R−T

The SE_T_, the SE value resulting from absorption (SE_A_), and the SE contributing by reflection (SE_R_) are calculated by Equations (8)–(10) [23,24]:

(8) $${SE}_{A}\left(dB\right)=-10\log\left(\frac{T}{1-R}\right)$$

(9) $${SE}_{R}\left(dB\right)=-10\log\left(1-R\right)$$

(10) $${SE}_{T}\left(dB\right)={SE}_{A}+{SE}_{R}+{SE}_{MR}$$

where SE_MR_ refers to the EMI SE contributed by multiple reflections of the incident electromagnetic waves. The SE_MR_ is negligible when the SE_T_ is larger than 10 dB because the multiply reflected waves are either absorbed or reflected by the EMI shielding material in this circumstance.

## 3. Results and Discussion

### 3.1. Morphologies

**Figure 2:**
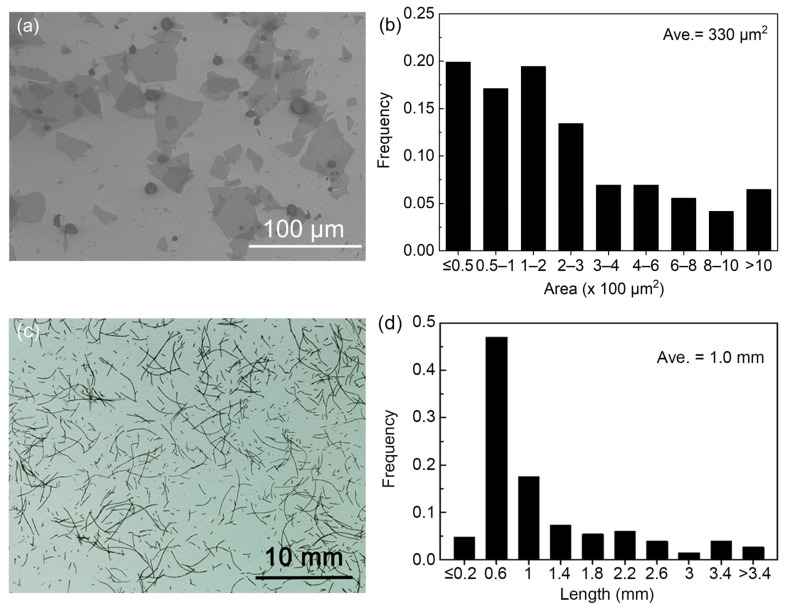
(**a**) SEM image of GO sheets used for fiber fabrication. (**b**) Size distribution of GO sheets obtained by analyzing SEM images by ImageJ. (**c**) Photo of fragmented GO fibers. (**d**) Length distribution of GO fiber obtained by analyzing photos by ImageJ.

The GO sheets utilized in the fabrication of fibers for nonwoven fabrics were meticulously characterized using SEM, as shown in Figure 2a. The as-purchased GO dispersion was synthesized via a modified Hummer’s method, which involves the oxidation and exfoliation of graphite [25]. To characterize whether the GO sheets are single-layered, we diluted GO dispersions to create a nearly transparent solution, which was subsequently deposited onto a SiO_2_/Si substrate through spin coating. The SEM images confirm that the GO sheets are predominantly single-layered (Figure 2a). Detailed size distribution analysis of the GO sheets was conducted using the ImageJ software, which processed the SEM images to quantify the dimensions of the sheets, as shown in Figure 2b. The analysis reveals a heterogeneous mixture of GO sheet sizes, encompassing both small and relatively larger sheets. The average area of these GO sheets is determined to be approximately 330 μm^2^, indicating a relatively large size. For the fabrication of nonwoven fabrics, wet-spun continuous fibers were then fragmented to generate short fibers, as shown in Figure 2c. The photograph highlights the variability in fiber lengths. The length distribution of these short GO fibers is further analyzed using ImageJ, which processes the photograph in Figure 2c. This analysis shows that the short fibers had an average length of 1.0 mm, as presented in Figure 2d.

**Figure 3:**
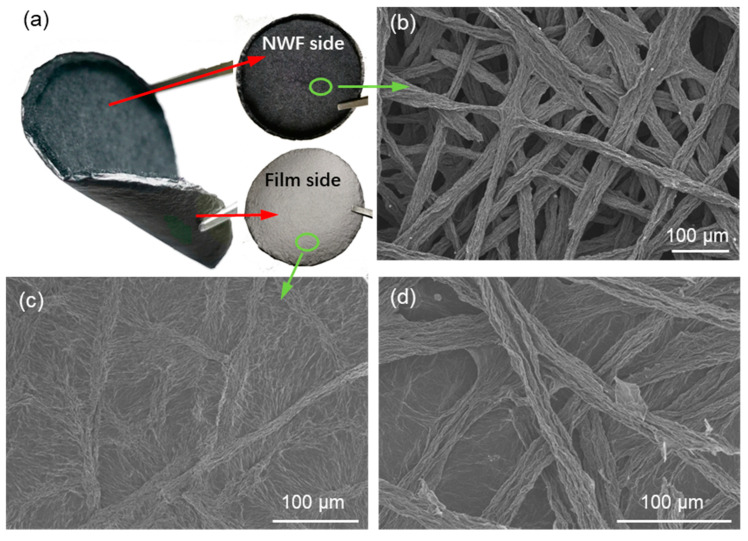
(**a**) Photographs of 2-gNWF. SEM images of (**b**) the fabric side, (**c**) the film side, and (**d**) the interfacial area of the two layers of 2-gNWF.

Morphologies of the 2-gNWFs were observed using both photographs and SEM, as shown in Figure 3. The 2-gNWF clearly shows a two-layer structure, with a looser upper layer and a denser lower layer (Figure 3a). Upon magnification and detailed observation through SEM, we obtained precise structural details of these two layers. The SEM image of the upper layer (Figure 3b) displays a clear fibrous structure where the fibers are interconnected, forming a nonwoven fabric. This indicates that the fragmented short GO fibers fused during the solvent removal process. The fusion process can be attributed to the driving effects of surface tension from the solvent and the Laplace pressure difference that occurs during the drying process [26]. In contrast, the lower layer of the 2-gNWF exhibits a film-like structure, as shown in Figure 3c. Although some fibers are faintly discernible, they have fused to form a dense, film-like layer. This structural formation results from the substantial pressure difference between the vacuum filtration bottle (the lower side of the filter membrane) and the short fibers suspended in the solvent (the upper side of the filter membrane). The vacuum filtration process, combined with the micro-pores in the filter membrane, causes the swollen GO fibers to rapidly aggregate on the filter membrane’s surface. These fibers are then compressed into a dense film by the pressure, thus forming the lower layer of the 2-gNWF. The formation of this dense film changes the pressure dynamics. Once the film covers the membrane pores, the pressure difference between the solvent and the film significantly decreases, allowing the upper layer to retain its fibrous structure. This phenomenon underscores the critical role of pressure dynamics and membrane characteristics in determining the final structure of the layers. To further investigate the interface between the two layers, we carefully peeled them apart and observed the structure at the interfacial region, as shown in Figure 3d. Observations at the interface revealed that fibers were either fused within the film or interconnected with the film, resulting in an integrated material.

**Figure 4:**
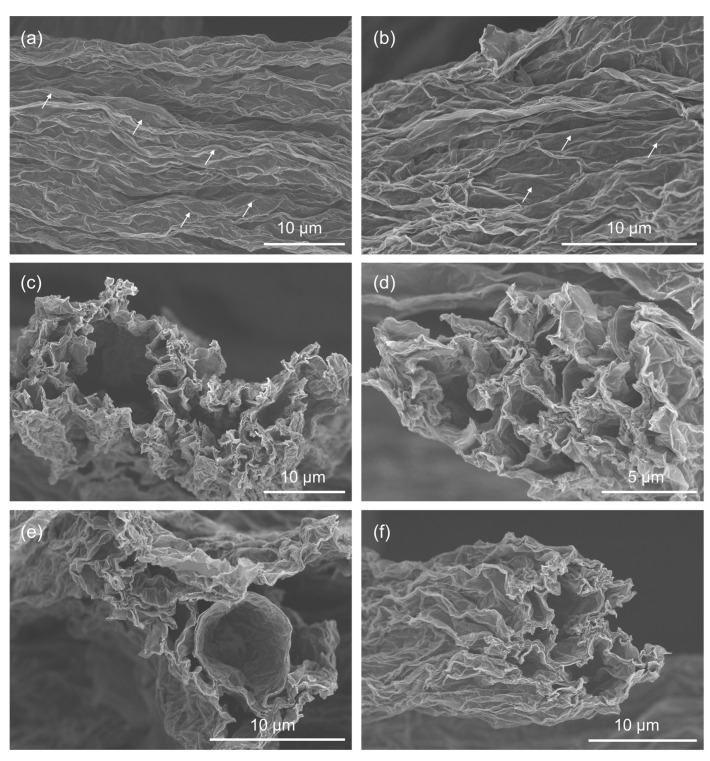
Typical SEM images of the (**a**,**b**) surfaces and (**c**–**f**) cross-sections of the fibers in the upper nonwoven fabric layer of 2-gNWF.

The detailed morphologies of the nonwoven fabric layer, characterized by a distinct fibrous structure, were further examined by SEM. Representative SEM images are presented in Figure 4. Post-reduction, the surfaces of the graphene fibers display pronounced wrinkles along their length, which is consistent with the morphologies observed in previously reported wet-spun graphene fibers [27,28]. In addition to the wrinkles, the presence of porous structures within the fibers can be inferred from the observation of bubbles, as identified by white arrows in Figure 4a,b. These bubbles are indicative of internal voids or pores, which are beneficial to EMI shielding applications. During the reduction process, particularly the thermal reduction process, oxygen-containing groups are reduced to form water molecules, resulting in porous structures of graphene fibers when releasing gaseous byproducts. To confirm the porous structure, cross-sectional SEM characterizations of the fiber layer in the 2-gNWF were conducted. Typical cross-sectional images are shown in Figure 4c–f. These images revealed highly porous fiber skeletons, characterized by a network of interconnected voids and channels. The combination of surface wrinkles and internal porosity in the graphene fibers indicates a complex hierarchical structure, which can enhance the performance of the nonwoven fabric in various applications.

**Figure 5:**
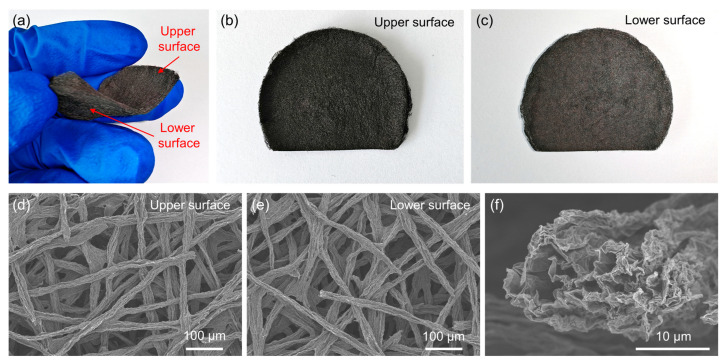
(**a**) Photograph of gNWF. Photographs of (**b**) the upper surface and (**c**) the lower surface of gNWF. SEM images of (**d**) the upper surface, (**e**) the lower surface, and (**f**) the cross-section of gNWF.

We also observed the morphologies of gNWFs using both photographs and SEM, as shown in Figure 5. Unlike the two-layer structure of 2-gNWFs, the gNWFs exhibit similar morphologies on both the upper and lower surfaces (Figure 5a–c). Detailed SEM analysis revealed that both surfaces of gNWFs display a nonwoven fabric structure (Figure 5d,e), which is similar to the fabric layer of 2-gNWFs. Additionally, the fibers in gNWFs exhibited porous structures (Figure 5f), also consistent with the fiber structure in the fabric layer of 2-gNWFs. Therefore, we can find that the structure of gNWFs is comparable to the fabric layer of 2-gNWFs.

### 3.2. EMI Shielding Properties

**Figure 6:**
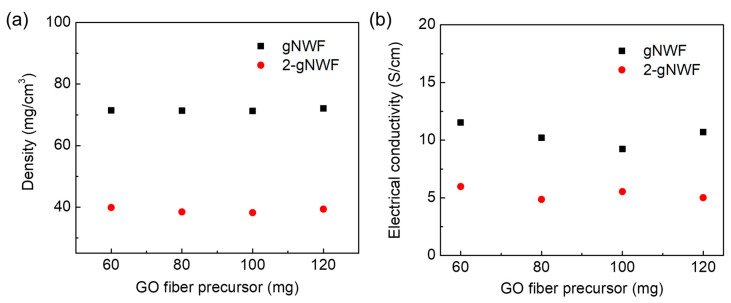
(**a**) Densities and (**b**) electrical conductivities of gNWFs and 2-gNWFs fabricated using different weights of GO fiber precursors.

The density of both gNWFs and 2-gNWFs fabricated using various weights of GO fiber precursors is shown in Figure 6a. It was found that the density of both gNWFs and 2-gNWFs remained consistent regardless of the weight of GO fibers used in their fabrication. Notably, the densities of 2-gNWFs (approximately 39 mg/cm^3^) are significantly lower than those of gNWFs (approximately 71 mg/cm^3^). This difference can be attributed to the smaller pressure difference during the vacuum filtration process for 2-gNWFs after the formation of the film layer, which results in a less dense structure. Additionally, the observation that both gNWFs and 2-gNWFs, which were fabricated using different weights of GO fibers, exhibit similar densities indicates that the density of these NWFs is independent of the initial weight of the GO fibers. This suggests a level of consistency in the fabrication process that is not influenced by the amount of precursor material used. Moreover, the electrical conductivities of the NWFs are also found to be independent of the weight of the GO fibers (Figure 6b). The gNWFs exhibited a higher electrical conductivity compared to 2-gNWFs, with average values of approximately 10 S/cm and 5 S/cm, respectively. The higher electrical conductivity of gNWFs can be primarily attributed to their greater density, which likely results in better contact between the graphene sheets and a more continuous conductive network. The denser gNWFs provide a more efficient pathway for electron transport, thereby enhancing their electrical conductivity.

**Figure 7:**
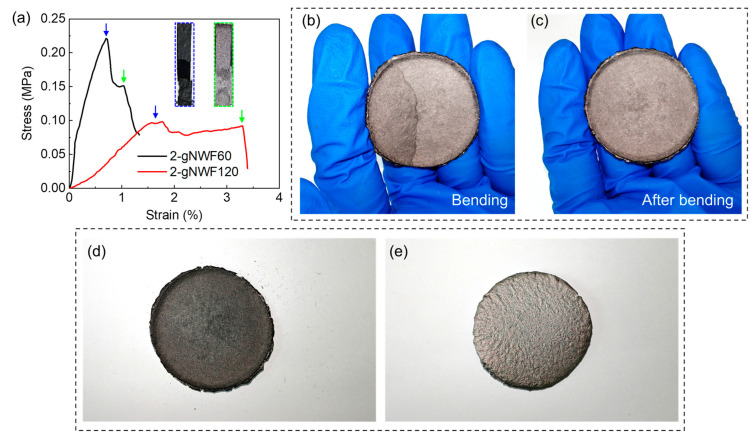
(**a**) Stress–strain curves of two typical 2-gNWFs during uniaxial tensile tests. Blue arrows indicate the breakage of the film-like layer, with the corresponding photograph shown in the blue dashed inset. Green arrows indicate the breakage of the fibrous upper layer, with the corresponding photograph shown in the green dashed inset. Photographs of 2-gNWF (**b**) during bending and (**c**) after releasing. Photographs of 2-gNWF after manual friction of (**d**) the fibrous upper layer and (**e**) the film-like lower layer.

The mechanical properties and stability of 2-gNWFs are shown in Figure 7. The stress–strain curves of two typical 2-gNWFs, 2-gNWF60 and 2-gNWF120, exhibit a peak value followed by a shoulder (Figure 7a). Due to the relatively dense structure of the film-like layer in 2-gNWFs, this layer is stronger but more brittle than the fibrous layer, leading to earlier breakage during uniaxial tension (as depicted in the blue dashed inset in Figure 7a) and the first peak stress (indicated by blue arrows in Figure 7a). After the breakage of the film-like layer, the fibrous layer can be further stretched, resulting in a shoulder in the stress–strain curve before complete breakage (green arrows and green dashed inset in Figure 7a). The 2-gNWF60 exhibits a tensile strength of approximately 0.22 MPa and an elastic modulus of around 68 MPa, while the 2-gNWF120 shows lower strength (around 0.1 MPa) and modulus (about 4 MPa), due to the thicker, weaker fibrous layer in 2-gNWF120. The 2-gNWFs demonstrate certain flexibility, showing a crease mark upon bending (Figure 7b) but recovering after release (Figure 7c). Furthermore, the structural stability of 2-gNWFs upon friction is also acceptable (Figure 7d,e). After manual friction on the fibrous layer of 2-gNWFs 100 times, only a small number of fibers were removed (Figure 7d). When friction was applied to the film-like layer, this layer remained intact and did not detach from the 2-gNWFs (Figure 7e). These results indicate that 2-gNWFs possess decent mechanical stability, making them suitable for EMI shielding applications.

**Figure 8:**
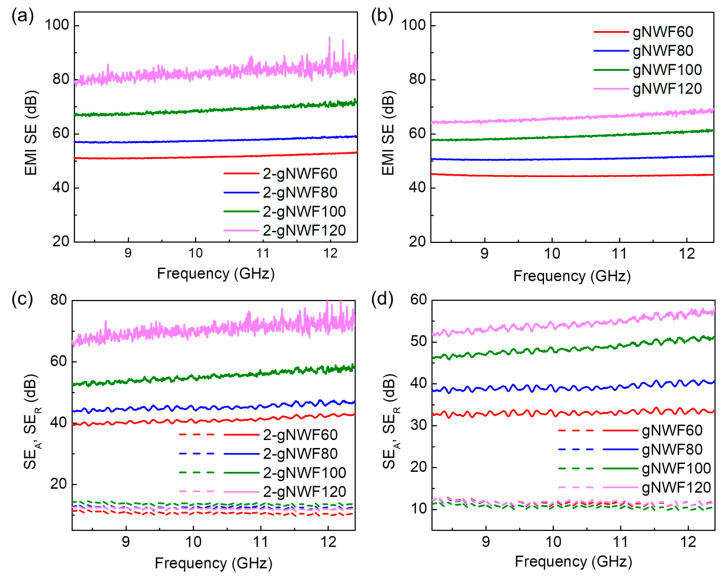
(**a**) EMI SE of 2-gNWFs. (**b**) EMI SE of gNWFs. (**c**) EMI SE_A_ and SE_R_ of 2-gNWFs. (**d**) EMI SE_A_ and SE_R_ of gNWFs. Note that the solid curves in (**c**,**d**) are SE_A_ of 2-gNWFs and gNWFs, while the dashed curves are SE_R_.

We further evaluated the EMI shielding properties of 2-gNWFs and gNWFs, as shown in Figure 8. These nonwoven fabrics were fabricated using different weights of GO fibers, denoted as 2-gNWFx and gNWFx, where x represents the weight of GO fibers used. For example, 2-gNWF60 refers to the two-layer graphene nonwoven fabric fabricated with 60 mg GO fibers. The EMI SE of the 2-gNWFs increases with the amount of GO fiber precursors used (Figure 8a). Specifically, the EMI SE for 2-gNWF60 is approximately 50 dB, and it increases to around 80 dB for 2-gNWF120 in the frequency range of 8.2–12.4 GHz (the X-band). Similarly, the EMI SE of gNWFs also improves with the use of larger amounts of GO fibers during fabrication (Figure 8b). For instance, gNWF60 exhibits an EMI SE of around 45 dB, which increased to approximately 64 dB for gNWF120 within the same frequency range. Given that the density of both 2-gNWFs and gNWFs fabricated with different weights of GO fibers remains consistent (Figure 6a), the use of greater weights of GO fiber precursors results in increased thickness of the NWFs. This increase in thickness is a significant factor contributing to the enhanced EMI shielding properties, as thicker materials generally provide better attenuation of electromagnetic waves [29,30]. Additionally, the EMI SE of 2-gNWFs is higher than that of the corresponding gNWF counterparts within the X-band range. Since the 2-gNWFs exhibit lower densities and greater thickness compared to gNWFs, the difference in EMI SE between these two materials can be primarily attributed to the difference in their thickness and a more porous structure [31]. The two-layer structure of 2-gNWF, with its looser upper layer and denser lower layer, may also contribute to the improved EMI shielding performance by providing additional interfaces for the reflection and absorption of electromagnetic waves.

To evaluate the contributions of absorption and reflection of incident electromagnetic radiations to the EMI SEs of 2-gNWFs and gNWFs, their SE values due to absorption (SE_A_) and reflection (SE_R_) are summarized in Figure 8c,d. The analysis reveals that the EMI shielding properties of both 2-gNWFs and gNWFs are predominantly attributed to the absorption of electromagnetic radiation. Specifically, the EMI SE_A_ values range from approximately 40 to 70 dB for 2-gNWFs and from approximately 33 to 55 dB for gNWFs in the X-band frequency range. These high SE_A_ values indicate that both types of nonwoven fabrics are highly effective at absorbing electromagnetic waves, which is a critical factor for effective EMI shielding materials. In contrast, the SE_R_ for both types of nonwoven fabrics is significantly lower, remaining below 15 dB. This disparity between SE_A_ and SE_R_ suggests that the primary mechanism for EMI shielding in these graphene-based materials is absorption rather than reflection. The absorption mechanism is typically more desirable for EMI shielding materials because it minimizes the reflection of electromagnetic waves back into the environment, thereby reducing potential interference with other electronic devices [32]. Furthermore, as the amount of GO fibers used for the fabrication of the fabrics increases, the SE_A_ of both 2-gNWFs and gNWFs exhibit significant increments. For example, 2-gNWF60—fabricated with 60 mg of GO fibers—shows an EMI SE_A_ of around 50 dB, which increased to approximately 80 dB for the 2-gNWF120 that was fabricated with 120 mg of GO fibers. Similarly, the EMI SE_A_ for gNWFs increases with the amount of GO fibers, reflecting a consistent enhancement in absorption capability with greater precursor material. However, the SE_R_ of these nonwoven fabrics shows only a marginal increase with the increasing GO fiber content. The relatively low and less variable SE_R_ values indicate that reflection is not significantly influenced by the amount of GO fibers, highlighting that the structural properties of the fabrics, such as thickness and density, play a more crucial role in determining absorption capabilities [33].

**Figure 9:**
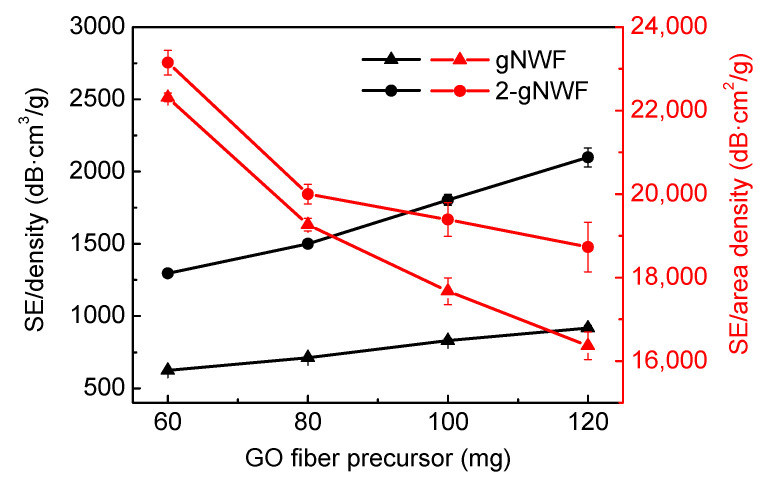
Specific EMI SEs of gNWFs and 2-gNWFs normalized by the density and area density.

The specific EMI SEs normalized by the density and area density are shown in Figure 9. When normalized by density, the specific EMI SE values of both 2-gNWFs and gNWFs show an increasing trend with the use of more GO fiber precursors, due to the increasing EMI SE values while maintaining similar densities. The specific EMI SE normalized by density for 2-gNWF120 reaches approximately 2000 dB cm^3^/g, indicating that the material is highly efficient in providing EMI shielding relative to its mass, which is particularly advantageous for applications where weight is a critical factor. On the other hand, when the specific EMI SE values are normalized by area density, both 2-gNWFs and gNWFs show a decreasing trend with increasing amounts of GO fiber precursors. The highest specific EMI SE value normalized by area density was observed for 2-gNWF60, which achieved around 23,000 dB cm^2^/g. This indicates that for a given surface area, the 2-gNWF60 sample is more efficient at EMI shielding compared to samples with higher amounts of GO fibers.

**Figure 10:**
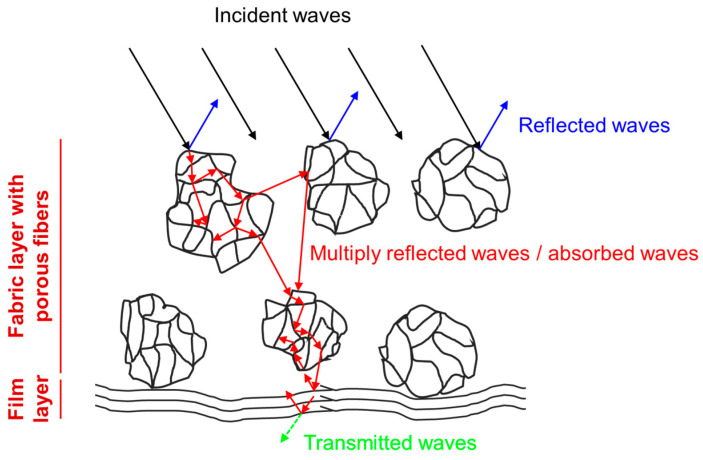
Schematic illustration of EMI-shielding mechanisms of 2-gNWF (cross-sectional view).

Based on the above results, the EMI shielding mechanism of 2-gNWF is shown in Figure 10. When incident electromagnetic waves reach 2-gNWF, they encounter highly conductive surfaces. The interaction between incident waves (black arrows in Figure 8) and free charges on the surface results in the reflection of some waves (blue arrows in Figure 8), contributing to the SE_R_. However, given the absorption-dominated EMI shielding properties of 2-gNWF, only a small percentage of the incident waves are reflected, while a large proportion enters into the 2-gNWF. Within the material, the highly porous fibrous skeleton and the porous structure among the fibers facilitate multiple reflections of the electromagnetic waves [18,34], as identified by the red arrows in Figure 8. This multiple-reflection process significantly enhances the interaction of the waves with the material. During these interactions, localized currents are generated in the conductive network, and polarization/relaxation of dipoles and free charges occurs, leading to energy dissipation and absorption of the electromagnetic waves [18,35]. When the SE_A_ is larger than 10 dB, most of the multiply reflected waves are further absorbed [18]. Therefore, EMI SE_A_ values of both 2-gNWFs and gNWFs are attributed to both direct absorption and absorption of multiply reflected waves [18]. Additionally, the conductive film layer serves as an effective reflector for electromagnetic radiation, which contributes to a higher SE_R_ in 2-gNWF compared to gNWF (Figure 8c,d). This combination of mechanisms—reflection by the conductive surfaces and the film layer, multiple reflections within the porous structure, and effective absorption due to localized currents and dipole polarization—provides a comprehensive EMI shielding effect in 2-gNWF.

The EMI properties of 2-gNWF are compared with reported neat graphene-based materials, as summarized in Table 1. Reduced graphene oxide (rGO) films can achieve EMI SEs over 25 dB with thicknesses below 300 μm [17,36,37,38,39]; however, these films often have high densities [37]. Additionally, the EMI SEs of rGO films may not be exceptionally high when considering advanced applications [17,36,37,38]. In contrast, films fabricated using pristine graphene [40] and electrochemically exfoliated graphene [41] can achieve EMI SEs over 90 dB, primarily due to the excellent electrical conductivity of these low-defect graphene materials. However, the fabrication processes for these materials are relatively complex and costly, which can be a drawback for large-scale production and application. Graphene aerogels offer an advantage in terms of low densities, making them attractive for lightweight applications, while their EMI SEs are generally low to moderate [42,43,44]. High-quality graphene produced by chemical vapor deposition (CVD) methods shows promising results. CVD-grown nanoporous graphene [45] and graphene paper [46] can achieve EMI SEs over 80 dB, due to the superior quality of graphene produced by CVD, which ensures excellent electrical conductivity and structural integrity. However, the CVD process is intricate and expensive, which can pose challenges for widespread use. Laser-induced graphene exhibits a porous structure and can achieve an EMI SE of around 30 dB with a thickness of 0.3 mm [47]. In comparison, the 2-gNWF developed in this study demonstrates remarkable properties, showing a low density of 0.039 g/cm^3^ and a high EMI SE of 80 dB. This performance is superior to a significant portion of neat graphene-based materials, showcasing the advantages of the two-layer nonwoven fabric structure. The combination of low density and high EMI SE in the 2-gNWF is particularly noteworthy. It indicates that the material can provide effective EMI shielding without adding significant weight, making it ideal for applications in aerospace, automotive, and portable electronics, where both weight and shielding performance are crucial considerations.

## 4. Conclusions

In this study, we have successfully developed a two-layer graphene-based nonwoven fabric (2-gNWF) that exhibits outstanding EMI shielding properties. The unique structural configuration of the 2-gNWF, comprising a highly porous fibrous upper layer and a dense conductive film-like lower layer, significantly enhances its shielding effectiveness. The 2-gNWF demonstrates an impressive EMI SE of 80 dB while maintaining a remarkably low density of 0.039 g/cm^3^, outperforming many existing graphene-based materials. Our study reveals that the excellent EMI shielding performance of 2-gNWF results from its ability to interact with electromagnetic waves predominantly through absorption, complemented by reflection and multiple internal reflections. The dense film layer further enhances the reflection component, contributing substantially to the overall shielding effectiveness. The innovative structure and exceptional performance of 2-gNWF highlight its potential to meet the increasing demand for effective and lightweight EMI shielding solutions in advanced technological applications.

## Figures and Tables

**Table 1:** Comparison of EMI SEs in the X-band range of different graphene-based materials.

Material	Electrical Conductivity (S/cm)	Density (g/cm^3^)	Thickness (mm)	EMI SE (dB)	SE/Density (dB·cm^3^/g)	SE/Area Density (dB cm^2^/g)	Ref.
rGO film	117	-	0.1	~60	-	-	[36]
rGO film	1780	0.91	0.013	~43	~47	~36,348	[37]
Porous graphene film	20.2	0.075	~0.2	43.8	640	29,178	[38]
rGO foam	3.1	0.06	0.3	25.2	420	14,000	[17]
Graphene aerogel film	2500	0.41	0.12	~75	183	15,243	[39]
Pristine graphene film	1340	1.49	0.1	~90	~67	~6700	[40]
Electrochemically exfoliated graphene film	600	-	0.125	108	-	93,358	[41]
Graphene aerogel	1.8	-	2.5	43	-	-	[42]
Graphene aerogel	0.01	0.006	2.5	27.6	4600	18,400	[43]
Honeycomb porous graphene	4	0.0388	0.005	~40	~1030	~206,000	[44]
CVD-grown nanoporous graphene	~33	0.045	0.03	83	1844	616,300	[45]
CVD-grown graphene paper	680	0.81	0.1	~100	~119	11,900	[46]
Laser-induced graphene	~10	-	~0.3	~30	-	-	[47]
2-gNWF	~5	0.039	1.12	~80	2000	23,000	This work

## Data Availability

Data are contained within the article.
